# A systematic literature review on first aid provided by laypeople to trauma victims

**DOI:** 10.1111/j.1399-6576.2012.02739.x

**Published:** 2012-08-17

**Authors:** T D Tannvik, H K Bakke, T Wisborg

**Affiliations:** 1 Department of Anaesthesiology and Intensive Care, Hammerfest HospitalHammerfest, Norway; 2 Anaesthesia and Critical Care Research Group, Faculty of Health Sciences, University of TromsøTromsø, Norway

## Abstract

Death from trauma is a significant and international problem. Outcome for patients suffering out-of-hospital cardiac arrests is significantly improved by early cardiopulmonary resuscitation. The usefulness of first aid given by laypeople in trauma is less well established. The aim of this study was to review the existing literature on first aid provided by laypeople to trauma victims and to establish how often first aid is provided, if it is performed correctly, and its impact on outcome. A systematic review was carried out, according to preferred reporting items for systematic reviews and meta-analysis (PRISMA) guidelines, of all studies involving first aid provided by laypeople to trauma victims. Cochrane, Embase, Medline, Pubmed, and Google Scholar databases were systematically searched. Ten eligible articles were identified involving a total of 5836 victims. Eight studies were related to patient outcome, while two studies were simulation based. The proportion of patients who received first aid ranged from 10.7% to 65%. Incorrect first aid was given in up to 83.7% of cases. Airway handling and haemorrhage control were particular areas of concern. One study from Iraq investigated survival and reported a 5.8% reduction in mortality. Two retrospective autopsy-based studies estimated that correct first aid could have reduced mortality by 1.8–4.5%. There is limited evidence regarding first aid provided by laypeople to trauma victims. Due to great heterogeneity in the studies, firm conclusions can not be drawn. However, the results show a potential mortality reduction if first aid is administered to trauma victims. Further research is necessary to establish this.

Trauma is a significant and international problem, causing approximately 1 out of every 10 deaths globally. It largely affects young people. In the age group 15–59, it is responsible for 13–29% of all deaths.^1^ Many trauma deaths occur in the pre-hospital setting (20–86%).^2^–^6^ Many patients succumb even before health-care personnel reach the scene, particularly in rural areas where there are long response and transport times for emergency medical services (EMS).^5^–^7^ In non-traumatic cardiac arrest, the actions of laypeople have been studied extensively, and it has been demonstrated that early and effective cardiopulmonary resuscitation improves the outcomes for patients who suffer out-of-hospital cardiac arrests.^8^–^21^ First aid in trauma has attained less attention; however, it has been postulated that a proportion of trauma deaths could be prevented by basic first aid measures at the scene.^9^, ^10^ It is probable that laypeople present at the scene of trauma can improve outcome by providing measures such as a free airway, stopping external bleeding, and preventing hypothermia. The aim of this study was therefore to review the existing literature on first aid provided by laypeople in face of injury, and thereby establish (1) how frequently first aid is carried out, (2) if it is performed correctly, and (3) its impact on outcome.

## Materials and methods

This review was conducted in accordance with the preferred reporting items for systematic reviews and meta-analysis (PRISMA) guidelines for systematic reviews.^11^

### Study sample

All studies concerned with lifesaving first aid performed by laypeople in pre-hospital trauma were eligible. First aid performed by professionals or by individuals with extensive first aid training such as military medics, or voluntary ambulance service personnel were excluded. Any original article or study, ranging from randomised controlled trials to population surveys, was considered for inclusion as existing research on the topic is sparse. Any time period and language was considered eligible. Inclusion and exclusion criteria are given in Table 1.

**Table 1:** The exclusion and inclusion criteria of the review

Inclusion criteria	Exclusion criteria
Trauma and first aid in the pre-hospital setting	Animal studies
	Duplicate articles
	Cardiac arrest due to non traumatic causes
Any language	
Any journal and publication date	First aid given by medical professionals or other highly trained personnel
All study types	
	Intra-hospital procedures
	Psychological trauma
	Isolated ocular trauma
	Minor burns
	Isolated dental trauma
	Near drowning

### Search strategy

The peer-reviewed databases Cochrane, Embase, Medline, and Pubmed were systematically searched as well as the non-peer-reviewed database Google Scholar, all from the first available record until July 2011. A broad scope was kept in the search to avoid omitting relevant papers on the topic. Search terms were (1) *trauma* or *high energy trauma* or *severe trauma* or *multitrauma* or *polytrauma* or *motor vehicle accident* or *car crash* or *train crash or catastrophe* or *crisis* or *sport injury* or *injury* or *pre-hospital care triage* AND/OR (2) *first aid* or *first responder* or *bystander* or *lay person.* The search items were systematically combined using the medical subject headings (MeSH) function of the various databases. The reference lists of the potentially eligible papers were also examined to identify papers that may have been overseen by the electronic search. The only limits applied to the search were that papers had to be relating to humans only and that they should contain an abstract with the search terms included.

### Study selection

One author screened all titles from the search, accessing abstracts when necessary and identified potentially eligible articles. Two authors independently examined the resulting abstracts. All articles selected by at least one of the authors were subjected to full-text review.

### Data extraction

The main details from each study retrieved for full-text analysis was recorded into a standardised form by the author assessing the study. The articles were then presented and discussed within the group.

### Outcome measures

The outcome measures that were considered were

- frequency of first aid,
- quality of first aid, and
- impact on outcome.

## Results

The result of the initial search yielded 2695 references. Many of the same articles were identified by the different databases, which resulted in a considerable overlap. In the first screening, 2663 references were excluded in clear concordance with the inclusion and exclusion criteria. The abstracts of the remaining 32 papers were reviewed, and further 12 papers were excluded. Full text versions of the remaining articles were then obtained and read by the group. One article was added, as it appeared on the reference list of one of the articles retrieved in full text. After careful consideration of the full text articles, further 11 articles were excluded. This resulted in 10 articles, which were finally deemed eligible for review. This process is illustrated in Fig..

**Fig. 1:**
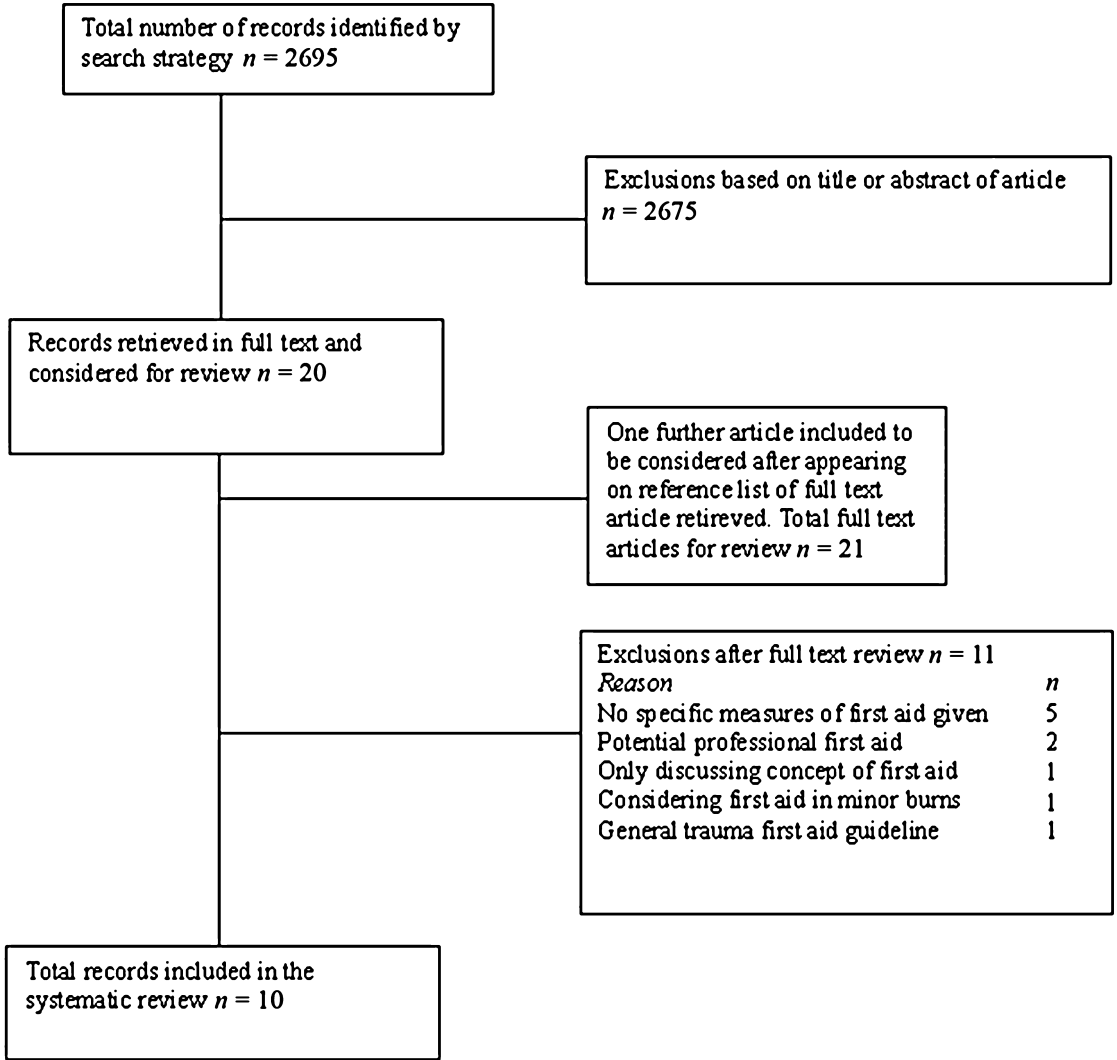
Illustration of the selection process for articles included in the review.

### Study design and quality

The included studies consisted of one cohort study, two simulation-based randomised control trials, three cross-sectional surveys, and four cross-sectional studies. Table 2 shows the involved studies, sample size, and the setting where the study was performed. The largest population studied was 11334^12^, however the total number of actual victims, in all included studies, was 5836, this is largely due to a study involving 2932 victims^13^, ^14^. It is of note that one study used the same study data to address different aspects of bystander first aid; we have therefore treated the two as one study.^13^, ^14^ The bias risk was considered individually in each of the included studies. The authors were contacted in those studies where the information required was not clearly stated or where there were uncertainties in regard to the methodology of the study.

**Table 2:** Information on the included studies showing author, type and size of study, setting, and country

Study by first author	Type of study	Number of participants	Setting	Country
Ashour et al.^15^	Cross-sectional study	112	Urban and rural	Australia
Ertl and Christ^19^	Randomised control trial	101	Simulation based	Germany
Henriksson et al.^21^	Cross-sectional study	474	Rural	Sweden
Khorasani-Zavareh et al.^16^	Cross-sectional survey	292	Rural	Iran
Macharia et al.^17^	Cross-sectional survey	310	Urban and rural	Kenya
Murad and Husum^20^	Cohort study	1341	Rural	Iraq
Nguyen et al.^12^	Cross-sectional survey	75	Urban	Vietnam
Pelinka et al.^13^*	Cross-sectional study	2932	Urban	Austria
Shotland and Heinold^18^	Randomised control trial	163	Simulation based	USA
Thierbach et al.^14^*	Cross-sectional study	2932	Urban	Austria

* Shared study material.

Five of the articles presented the incidence of bystander first aid as a percentage of the total number of situations where first aid was appropriate, as seen in Table 3.^12^, ^15^–^18^ The frequency of any kind of first aid in these studies ranges from 10.7% to 65%. Three studies gave information on the type of first aid provided,^13^, ^14^, ^18^, ^19^ and the most important findings of these studies are shown in Table 4. The presence of bystanders was assessed by one of the studies, which showed a bystander presence in 59% of cases.^13^, ^14^

**Table 3:** Frequency and setting of first aid given by laypeople to trauma victims

Study by first author	Frequency of first aid (%)
Ashour et al.^15^	10.7% given first aid in fatal traffic-related trauma
Khorasani-Zavareh et al.^16^	65% given first aid in traffic-related trauma
Macharia et al.^17^	16% given first aid in traffic-related trauma
Nguyen et al.^12^	41% given first aid in traffic-related trauma
Shotland and Heinold^18^	22% given first aid in simulation of arterial bleed

**Table 4:** Frequency of specific first aid measures given by laypeople to trauma victims

Study by first author	Airway handling	Use of recovery position (%)	Control of bleeding (%)	Prevention of hypothermia (%)
Ertl and Christ^19^	Airway check 11.5%	63.5	55.8	44.2
	Head tilt jaw thrust 26.9%			
	Checks breathing 59.6%			
Shotland and Heinold^18^	–	–	22	–
Pelinka et al.^13^ and Thierbach et al.^14^	No airway handling specified except use of recovery position.	73	60	42

Three studies gave information on the adequacy of the first aid given.^13^, ^14^, ^18^, ^19^ One study investigated the impact of assistance from a mobile multimedia device on quality of first aid through a randomised controlled trial where laypeople performed first aid in two simulated scenarios.^19^ Due to the exclusion criteria, only the first scenario of this study, which concerned haemorrhage control, was eligible for inclusion. The control group of this scenario, which were laypeople receiving no help, was eligible for inclusion in this review. Another study investigated college students' response to an unsuspected simulation of an arterial bleed, which was interpreted as real by 96.3% of the participants.^18^ The responses to the college students were then noted and included in the study. The two studies that shared study data had EMS personnel assessing the quality of the first aid given to actual patients, and reported the findings according to the first aider's level of training.^13^, ^14^ A summary of these findings are seen in Table.

**Table 5:** Frequency of incorrect first aid measures given by laypeople to trauma victims

Study by first author	Use of recovery position (%)	Control of bleed (%)	Prevention of hypothermia (%)
Ertl and Christ^19^	–	83.7	–
Pelinka et al.^13^ and Thierbach et al.^14^*	1–11	4–9	0–13

* Depending on the bystander's level of training.

There was one single study that investigated the impact of first aid on mortality, and it found a 5.8% decrease when first aid was provided.^20^ Two autopsy-based studies estimated a potential decrease in mortality, by 4.5% and 1.8%, had first aid been carried out.^15^, ^21^ The two studies underline the importance of controlling bleeding and providing a free airway to the trauma victim.^15^, ^21^

## Discussion

Research regarding first aid provided by laypeople in trauma situations is sparse. This review found considerable variability in both the frequency and the quality of first aid provided by laypeople. There is, however, some theoretical and quantitative support for the concept that early and effective first aid in trauma situations may improve the survival of trauma victims. The frequency of bystanders providing first aid in the included studies ranged from 10.7% to 65%. It should be noted that the study with the lowest intervention rate^15^ only investigated trauma fatalities and hence not necessarily reflects the intervention rate for non-fatal trauma. However, the study with the second lowest intervention rate^17^ had no such limitation and a first aid rate of 16%. Factors that may contribute to the large variability of the frequency of first aid could include severity of the injury, fear, inability to recognise injury, or expectation of emergency services to arrive. Only one of the studies^13^, ^14^ mentioned the presence of bystanders, and reported bystander presence in 59% of cases. To fully appreciate the actual initiative of the bystanders to give first aid, one needs to establish how often bystanders are in fact present. Whether first aid is performed correctly was investigated by three studies. In a controlled scenario where the participants had a clear expectation to carry out first aid, incorrect handling of arterial bleeding was reported to be 83.7% of the participants in the control group not actively assisted during the scenario.^19^ An ambulance-based study reported that first aid was performed incorrectly in 0–13% of cases depending on the specific measure and bystander's level of training.^13^, ^14^ The quality of first aid was judged according to local ambulance service guidelines. None of these studies indicate whether incorrect first aid was harmful or potentially harmful to the patient. Factors such as cold, rain, multiple casualties, emotional stress, and poor light conditions would likely have a negative impact on performance. It is therefore unexpected that the rate of incorrect first aid was substantially higher in a clearly laboratory-based setting compared with the ‘real-life’ scenarios and a more realistic experimental scenario.^13^, ^14^, ^18^ In the laboratory-based scenario, the participants were expected to perform first aid, and this expectation could influence the participants to provide first aid measures with which they are unfamiliar with, where in a real-life situation, they would refrain from acting. Rigid assessment criteria may also contribute to the discrepancy between a laboratory-based study and the studies involving actual patients. The EMS personnel, in the real-life study, were assessing the first aid in addition to providing medical assistance to the injured and therefore may have been less critical of the bystander's performance.^13^, ^14^ On the other hand, a dressing deemed incorrect in the laboratory-based scenario may well be sufficient to staunch a bleeding in real life, as the ambulance study included any bleeding and not just arterial injury.^13^, ^14^, ^19^

A decrease in mortality was assessed in one of the studies,^20^ where first aid gave a 5.8% reduction of mortality compared with victims who received no first aid. However, this study was performed in a war zone with a high rate of penetrating trauma (42%). As penetrating trauma is more likely than blunt trauma to result in external bleeding, the survival benefit of first aid by laypeople is predicted to be lower in situations of blunt trauma. Therefore, this result may not be generalisable to areas where blunt trauma is predominant.

The possible mortality reduction through bystander first aid in blunt trauma has been estimated by two autopsy-based studies.^15^, ^21^ These studies obtained data from ambulance records, hospital records, and autopsy records of fatal motor vehicle accidents. In addition, one of the two studies^21^ also used police records and toxicology reports. The estimated reductions in mortality were 4.5% and 1.8%, respectively. Each of these studies had sound methodology and good internal validity, and the estimated reduction in mortality is therefore likely a realistic reflection of the benefit of first aid in fatal blunt trauma. The key first aid measures that were identified as potentially life saving were the provision of a free airway and the control of external bleeding.

There was a great heterogeneity among the included studies, and hence a meta-analysis could not be performed. In particular, the three interview-based surveys^12^, ^16^, ^17^ carry large bias risks. Five databases were included in this search, and only articles that had an abstract in English could be identified by the search strategy. Hence, some studies could have been missed. The included studies originate from very different settings, stretching from war zones in Iraq to simulation-based scenarios at western universities. The organisation of health care, EMS, and general economic state of the countries also greatly vary between these studies. These factors confirm the lack of studies on this particular topic and make it impossible to draw any firm conclusions on the results gathered across several studies. We are satisfied that the broad scope of the literature search and thorough review of abstracts ensured that the likelihood of overlooking relevant articles was minimised.

Perhaps the low number of studies and wide range of results also reveals that the field itself is very diverse and difficult to study. When taking into consideration the large burden that trauma represents, there is still a large need for further research into the topic. The results indicate that there is a potential in increased focus on layperson first aid, and that improvement is likely to be worthwhile. The available evidence supports that correct bystander first aid is likely to have an effect on mortality in trauma. Further research should try to establish to what extent first aid is given in relation to the presence of bystanders, if it is correct, and what specific measures are in need of attention with a particular focus on airway handling and haemorrhage control. It would also be pertinent to ask to what extent the general population in a future study is taught first aid. The need for further high-quality studies may be of extra importance in rural and remote areas of the industrialised world, with prolonged EMS response times, and in the third world due to lack of professional pre-hospital services.
